# Blockade of 4-1BB and 4-1BBL Interaction Reduces Obesity-Induced Skeletal Muscle Inflammation

**DOI:** 10.1155/2013/865159

**Published:** 2013-12-18

**Authors:** Ngoc Hoan Le, Chu-Sook Kim, Thai Hien Tu, Hye-Sun Choi, Byung-Sam Kim, Teruo Kawada, Tsuyoshi Goto, Taesun Park, Jung Han Yoon Park, Rina Yu

**Affiliations:** ^1^Department of Food Science and Nutrition, University of Ulsan, Ulsan 680-749, Republic of Korea; ^2^Department of Biological Science, University of Ulsan, Ulsan 680-749, Republic of Korea; ^3^Graduate School of Agriculture, Kyoto University, Uji, Kyoto 611-0011, Japan; ^4^Department of Food and Nutrition, Yonsei University, Seoul 120-749, Republic of Korea; ^5^Department of Food Science and Nutrition and Research Institute for Bioscience & Biotechnology, Hallym University, Chuncheon 200-702, Republic of Korea

## Abstract

Obesity-induced skeletal muscle inflammation is characterized by increased macrophage infiltration and inflammatory cytokine production. In this study, we investigated whether 4-1BB, a member of the TNF receptor superfamily (TNFRSF9) that provides inflammatory signals, participates in obesity-induced skeletal muscle inflammation. Expression of the 4-1BB gene, accompanied by increased levels of inflammatory cytokines, was markedly upregulated in the skeletal muscle of obese mice fed a high-fat diet, in muscle cells treated with obesity factors, and in cocultured muscle cells/macrophages. In vitro stimulation of 4-1BB with agonistic antibody increased inflammatory cytokine levels in TNF*α*-pretreated muscle cells, and this effect was absent in cells derived from 4-1BB-deficient mice. Conversely, disruption of the interaction between 4-1BB and its ligand (4-1BBL) with blocking antibody decreased the release of inflammatory cytokines from cocultured muscle cells/macrophages. Moreover, deficiency of 4-1BB markedly reduced macrophage infiltration and inflammatory cytokine production in the skeletal muscle of mice fed a high-fat diet. These findings indicate that 4-1BB mediates the inflammatory responses in obese skeletal muscle by interacting with its ligand 4-1BBL on macrophages. Therefore, 4-1BB and 4-1BBL may be useful targets for prevention of obesity-induced inflammation in skeletal muscle.

## 1. Introduction

Obesity-induced inflammation, which is commonly observed in major metabolic organs such as adipose tissue, skeletal muscle, and liver, is characterized by increased accumulation of immune cells (macrophages, T cells) and inflammatory cytokine/chemokine production and plays a crucial role in the development of metabolic disorders such as insulin resistance and type 2 diabetes [1, 2]. It is likely that inflammation of skeletal muscle contributes to the diminished fatty acid oxidation and increased insulin resistance [3–7], leading to metabolic complications. Given that skeletal muscle, which constitutes up to 40–50% of body mass, is a major site regulating lipid and glucose metabolism [8], targeting skeletal muscle inflammatory components may be a useful strategy for protecting against obesity-related metabolic disorders. However, the factors involved in obesity-induced skeletal muscle inflammation remain unclear.

4-1BB (tumor necrosis factor receptor superfamily 9, TNFRSF9) is a costimulatory receptor mainly expressed on the surface of activated T cells and natural killer (NK) T cells [9, 10], while its ligand (4-1BBL, TNFSF9) is expressed on antigen-presenting cells such as macrophages [11]. The 4-1BB/4-1BBL interaction regulates various inflammatory processes such as cytokine production and immune cell proliferation/survival [11, 12]. Blockade of the 4-1BB/4-1BBL interaction using specific antibodies and/or knockout mice is beneficial in several inflammatory diseases (e.g., myocarditis, atherosclerosis, and sepsis) [13–15]. We have shown that obesity-induced adipose tissue inflammation is reduced by 4-1BB deficiency [16] and that this is due to disruption of the crosstalk between adipocytes and macrophages via the 4-1BB/4-1BBL interaction in adipose tissue [17]. Given that interaction between muscle cells and macrophages is important in obesity-induced skeletal muscle inflammation [5, 6], we hypothesized that 4-1BB and/or 4-1BBL might also play a role in the skeletal muscle inflammation in obesity.

In this study, we demonstrate that 4-1BB expression is upregulated in response to increasing inflammatory cytokine levels in the skeletal muscle of high-fat diet-fed mice, in muscle cells treated with obesity-related factors, and in cocultured muscle cells/macrophages. In addition, stimulation of 4-1BB enhances inflammatory cytokines in TNF*α*-pretreated skeletal muscle cells. Conversely, disruption of the 4-1BB/4-1BBL interaction reduces inflammatory responses in cocultured muscle cells/macrophages and in the skeletal muscle of obese mice fed a high-fat diet. 4-1BB and 4-1BBL may be useful targets for preventing obesity-induced inflammation in skeletal muscle.

## 2. Materials and Methods

### 2.1. Animals

The 4-1BB-deficient mice on a C57BL/6 background were established in the Immunomodulation Research Center of University of Ulsan, South Korea [18]. Male 4-1BB-deficient mice and their wild-type (WT) littermate controls at eight weeks of age were individually housed in plastic cages in a specific pathogen-free animal facility with a 12 h light, 12 h dark cycle. The mice were fed a high-fat diet (HFD) (60% of calories from fat; Research Diets Inc., New Brunswick, NJ, USA) or a regular diet (RD) (13% of calories from soybean oil; Harlan Teklad, Madison, WI, USA) for 9 weeks and given free access to food and water. All animal experiments were approved by the animal ethics committee of the University of Ulsan and conformed to National Institutes of Health guidelines. The animals were killed by CO_2_ asphyxiation and quadriceps muscles were dissected.

### 2.2. Antibodies

Agonistic monoclonal antibody against 4-1BB (3E1) was generated from ascites of nude mice [19]. The antibody (Ab) was purified from ascites by affinity column chromatography with protein G Sepharose (Sigma, MO, USA). Antagonistic monoclonal Ab against 4-1BBL (TKS-1) was purchased from e-Bioscience (San Diego, CA, USA). Rat immunoglobulin G (rat IgG) was purchased from Sigma and used as control.

### 2.3. Cell Culture

The mouse myoblast cell line C2C12 and the monocyte/macrophage-like cell line Raw264.7 were purchased from the American Type Culture Collection (ATCC, Manassas, USA). The C2C12 myoblasts (2-3 × 10^5^ cells/mL) were grown at 37°C in 5% CO_2_ in Dulbecco's modified Eagle's medium (DMEM) (Gibco, NY, USA) containing 10% fetal bovine serum (FBS) (Gibco), 100 units/mL penicillin, 100 *μ*g/mL streptomycin (Invitrogen, Carlsbad, CA, USA), and 20 *μ*g/mL gentamicin (Gibco). When the cells reached 100% confluence, the medium was replaced with the differentiation medium consisting of DMEM plus 2% horse serum (Gibco), which was changed after 2 days. Palmitic acid (Sigma) was dissolved in ethanol and conjugated with BSA at a 10 : 1 molar ratio before use. Lipopolysaccharide (LPS) (Sigma) was dissolved in water. Recombinant mouse TNF*α* (R&D Systems, Minneapolis, MN, USA) was reconstituted in PBS. After 3 days of differentiation, myotubes were incubated with 500 *μ*M palmitic acid (FFA) in serum-free DMEM containing 50 *μ*M BSA or with 10 ng/mL TNF*α*, or 100 ng/mL LPS in serum-free DMEM for 24 h. The equal amount of ethanol was used as control of FFA-treated group and the medium with no treatment was used as control of TNF*α*- and LPS-treated groups. Raw264.7 cells were cultured to 80% confluence at 37°C in 5% CO_2_ in RPMI medium (Gibco) containing 10% fetal bovine serum, 100 units/mL penicillin, 100 *μ*g/mL streptomycin (Invitrogen), and 20 *μ*g/mL gentamicin (Gibco). For coculture, the Raw264.7 cells were detached with 0.05% trypsin-EDTA (Gibco) and a number corresponding to 25% of the number of confluent myoblasts (8–10 × 10^5^ myoblasts/well of 24 well-plate) was directly seeded into culture plates containing 3 days differentiated myotubes (contact cocultures) in serum-free DMEM for 24 h. The transwell coculture system (Corning Incorporated, Corning, NY, USA) was used as control of the contact coculture. In the transwell coculture, the same number of Raw264.7 cells was added to transwell inserts (upper wells, 0.4 *μ*m pore size of membrane) to separate the cells from myotubes (lower wells). After incubation time, the culture media were collected for ELISA to determine the concentration of cytokines, and the cells were washed twice with PBS and lysed in Trizol Reagent (Invitrogen) for quantitative real-time PCR analysis.

### 2.4. Magnetic Separation of Myotubes and Macrophages

To see the effect of the contact coculture on each cell type, we separated myotubes and macrophages using a CD11b MicroBeads (MACS; Miltenyi Biotec, Sunnyvale, CA, USA). The cocultured cells were collected using 0.05% trypsin-EDTA (Gibco) and washed twice with washing buffer (PBS supplemented with 0.5% BSA and 2 mM EDTA). Subsequently, all 10^7^ total cells were incubated with 10 *μ*L CD11b MicroBeads for 15 min at 4°C. The cells were then washed, resuspended, and added to MACS column in a magnetic field, which retained CD11b-labeled cells and allowed myotubes to flow through. In a nonmagnetic field, macrophages were collected with an appropriate amount of washing buffer using a plunger supplied with the column. After isolation, a single population of cells was washed once with PBS and lysed in Trizol Reagent (Invitrogen) for RNA extraction.

### 2.5. Primary Mouse Skeletal Muscle Cells Isolation and Culture

Myoblasts were isolated from mouse hindlimbs as described previously [20]. Male C57BL/6 mice at 4 weeks of age were killed by CO_2_ asphyxiation. Briefly, the tibialis anterior muscle was collected and visible blood vessels and connective tissues were removed. Then, the muscle was minced in a solution of dispase II (Sigma) and collagenase D (Roche Molecular Biochemicals, Mannheim, Germany). The minced tissue was digested at 37°C for 30–45 min. The digested tissue was then filtered through a 100 *μ*m nylon mesh (SPL Lifescience, Pocheon, Korea). Subsequently, the filtered slurry was centrifuged for 5 min at 350 g and the pellet was resuspended in Ham's F10 (Gibco) containing 20% FBS and 2.5 ng/mL basis fibroblast growth factor (bFGF) (Sigma) and plated in a 60 mm collagen (Sigma) coated culture dish. The cells were incubated in an incubator at 37°C in 5% CO_2_, and the medium was changed every 2 days. At confluence of 50–60%, the cells were removed using PBS and the medium was then changed to F10/DMEM (ratio 1/1) plus 20% FBS and 2.5 ng/mL bFGF. When the cells reached confluence at 100%, the medium was replaced with the differentiation medium consisting of DMEM plus 2% horse serum (Gibco).

### 2.6. Quantitative Real-Time PCR (qRT-PCR)

Quadriceps muscle tissues were collected and stored at −20°C in RNAlater (Ambion, Austin, TX, USA). Total RNA was extracted from 50 mg muscle tissue samples or lysed cells with Trizol Reagent (Invitrogen). Two microgram aliquots of total RNA were reverse transcribed to cDNA using M-MLV reverse transcriptase (Promega, Madison, WI, USA). The qRT-PCR amplification of the cDNA was performed in duplicate with a SYBR premix Ex Taq kit (TaKaRa Bio Inc., Forster, CA, USA) using a Thermal Cycler Dice (TaKaRa Bio Inc., Japan). All reactions were performed with the same schedule: 95°C for 10 s and 40 cycles of 95°C for 5 s and 60°C for 30 s. Results were analyzed with Real Time System TP800 software (Takara Bio Inc.) and all values were normalized to the levels of the house-keeping gene **β**-actin. The primers used in the analysis are listed in Table 1.

### 2.7. Western Blot Analysis

Mice were killed by CO_2_ asphyxiation. Briefly, quadriceps muscle tissues were dissected and immediately frozen in liquid nitrogen. For protein extraction, the tissues and cell cultures were homogenized in lysis buffer containing 150 mM NaCl, 50 mM Tris-HCl, 1 mM EDTA, 50 mM NaF, 10 mM Na_4_P_2_O_7_, 1% IGEPAL, 2 mM Na_3_VO_4_, 0.25% protease inhibitor cocktail, and 1% phosphatase inhibitor cocktail (Sigma). The homogenates were centrifuged at 12000 g for 20 min at 4°C. The protein content was determined using a BCA Protein Assay Kit (Pierce, Rockford, IL, USA). Samples of 50 *μ*g or 10 *μ*g total protein extracted from tissue or cell culture, respectively, were subjected to western blot analysis using polyclonal antibodies to detect CD68, I**κ**B*α* (Santa Cruz Biotechnology, Santa Cruz, CA, USA), p-IKK*α*/**β**, and IKK**β** (Cell Signaling, Danvers, MA, USA). *α*-Tubulin was used as a loading control, measured with mouse *α*-tubulin antibody (Abcam, MA, USA).

### 2.8. Measurement of TNF*α*, IL-6, and MCP-1 Proteins

Quadriceps muscles (100 mg) were homogenized in 1 mL of 100 mM Tris-HCl and 250 mM sucrose buffer (pH 7.4) supplemented with 0.25% protease inhibitor cocktail (Sigma), and the pellets were removed by centrifuging at 10,000 g for 10 min at 4°C. Levels of TNF*α*, IL-6, and MCP-1 in the homogenates were measured by enzyme-linked immunosorbent assays (ELISA) using an OptEIA mouse TNF*α*/MCP-1 set (BD Biosciences, NJ, USA) and an IL-6 ELISA kit (R&D Systems, Minneapolis, MN, USA). Amounts of cytokine were adjusted for the protein contents of the homogenates determined with a BCA protein assay kit (Pierce).

### 2.9. Histological Analysis

Quadriceps muscles were fixed overnight at room temperature in 10% formaldehyde (Sigma) and embedded in paraffin. Immunohistochemical staining was performed with anti-CD68 (Santa Cruz Biotechnology). Secondary antibodies were anti-rabbit (Cell Signaling), and detection was with a Peroxidase Substrate kit (Vector Laboratories Inc., Burlingame, CA, USA).

### 2.10. Statistical Analysis

The results are presented as means ± standard error of the mean (SEM). Variables were compared using Student's *t*-test or analysis of variation (ANOVA) with Duncan's multiple-range test. Differences were considered significant at *P* < 0.05.

## 3. Results

### 3.1. Expression of 4-1BB/4-1BBL mRNAs in Obese Skeletal Muscle

**Figure 1:**
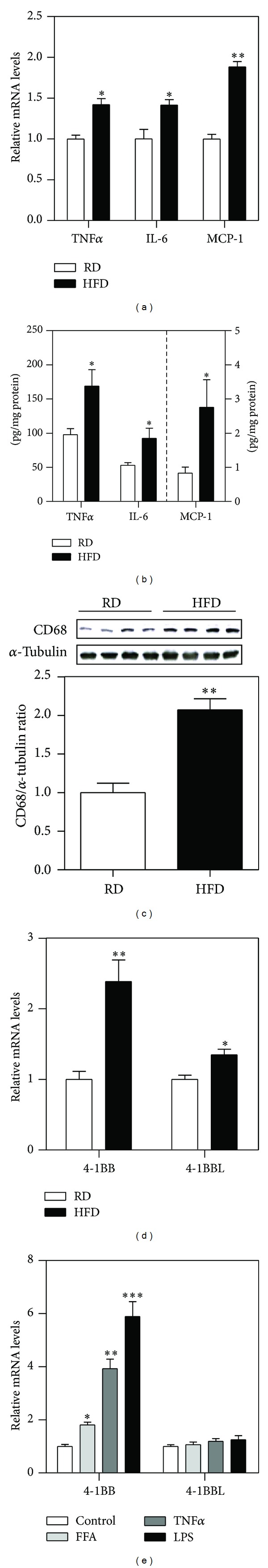
Levels of 4-1BB/4-1BBL mRNAs and inflammatory markers in obese skeletal muscle. C57BL/6 mice were fed a regular diet (RD) or high-fat diet (HFD) for 9 weeks. ((a) and (b)) TNF*α*, IL-6, and MCP-1 mRNAs and proteins were measured by qRT-PCR and ELISA, respectively. (c) Expression of CD68 and *α*-tubulin proteins was determined by western blotting using the indicated antibodies. (d) Expression of 4-1BB and 4-1BBL mRNAs in skeletal muscle was determined by qRT-PCR. Data are means ± SEM for *n* = 6. *P < 0.05, **P < 0.01 compared with RD group. (e) C2C12 myotubes were established for 3 days and then exposed to 500 *μ*M palmitic acid (FFA) in serum-free DMEM containing 50 *μ*M BSA, 10 ng/mL TNF*α*, or 100 ng/mL LPS in serum-free DMEM for 24 h. Ethanol was used as control of FFA-treated group and the medium with no treatment was used as control of TNF*α*- and LPS-treated groups. Expression of 4-1BB and 4-1BBL mRNAs in myotubes was determined by qRT-PCR. Data are means ± SEM of three independent triplicate experiments. *P < 0.05, **P < 0.01, and ***P < 0.001 compared with controls.

As shown in Figures 1(a) and 1(b), HFD feeding resulted in increased levels of inflammatory cytokines such as TNF*α*, IL-6, and MCP-1 at the mRNA and protein levels in muscle. Protein levels of the macrophage marker CD68 were also increased in the muscle of HFD-fed mice (Figure 1(c)). Levels of 4-1BB and 4-1BBL mRNAs in the muscles of HFD-fed mice were significantly higher than those in RD-fed mice (Figure 1(d)). We then examined the effects of obesity-related factors, including free fatty acids (e.g., palmitic acid), lipopolysaccharide (LPS), and cytokines (e.g., TNF*α*) on the expression of 4-1BB and 4-1BBL in C2C12 skeletal muscle cells.  Figure 1(e) shows that treatment with the obesity-related factors upregulated expression of 4-1BB but not 4-1BBL mRNA in C2C12 myotubes.

### 3.2. The 4-1BB/4-1BBL Interaction Enhances Inflammatory Cytokine Production in Myotubes/Macrophage Coculture

**Figure 2:**
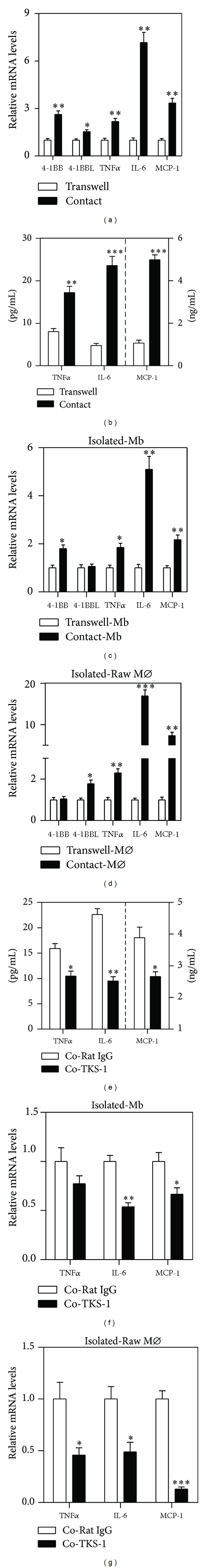
Inhibition of 4-1BB/4-1BBL interaction reduces inflammatory responses in myotube/macrophage coculture. C2C12 myotubes (Mb) were established for 3 days and then cocultured with Raw264.7 macrophages (M*⌀*) (25%) in serum-free DMEM for 24 h. ((a) and (b)) Raw macrophages were directly seeded into the plates containing established myotubes (Contact) or were added to transwell inserts (Transwell). (a) Expression of 4-1BB, 4-1BBL, and inflammatory cytokine (TNF*α*, IL-6, and MCP-1) mRNAs was analyzed by qRT-PCR. (b) TNF*α*, IL-6, and MCP-1 proteins released in the culture media were measured by ELSA. ((c) and (d)) Myotubes and Raw264.7 macrophages in the contact cocultures (Contact-Mb/M*⌀*) or the transwell cocultures (Transwell-Mb/M*⌀*) (control) were separated using a CD11b MicroBeads system. Expression of 4-1BB, 4-1BBL, and inflammatory cytokine mRNAs in isolated myotubes (c) and in isolated-Raw macrophages (d) was analyzed by qRT-PCR. (e–g) Raw264.7 macrophages were seeded onto myotubes with or without pretreatment with neutralizing anti-4-1BBL antibody (TKS-1, 5 *μ*g/mL) or rat IgG (5 *μ*g/mL) in serum-free DMEM for 24 h. (e) TNF*α*, IL-6, and MCP-1 proteins released in the culture media were measured by ELISA. ((f) and (g)) Myotubes and Raw264.7 macrophages in the TKS-1- or rat IgG-treated cocultures were separated using a CD11b MicroBeads system. Expression of inflammatory cytokine mRNAs in isolated myotubes (f) and in isolated-Raw macrophages (g) was analyzed by qRT-PCR. Data are means ± SEM of three independent triplicate experiments. *P < 0.05, **P < 0.01, and ***P < 0.001 compared with controls.

Because upregulation of 4-1BB expression in the muscle tissue of obese mice was accompanied by increased macrophage infiltration, we thought that the interaction of 4-1BB on muscle cells with its ligand 4-1BBL on macrophages might be responsible for the inflammation of obese skeletal muscle. To test this, we cocultured C2C12 myotubes with Raw264.7 macrophages in a direct contact coculture system or in a transwell coculture system and found that both 4-1BB and 4-1BBL mRNA expressions were significantly increased in the contact cocultures compared with the transwell cocultures (Figure 2(a)). We additionally confirmed that the contact coculture of muscle cells and macrophages resulted in elevated mRNA and protein levels of the inflammatory cytokines TNF*α*, IL-6, and MCP-1 (Figures 2(a) and 2(b)). To see the effect of the contact coculture on specific cell types, we separated myotubes and Raw macrophages from the cocultures and found that the expression of 4-1BB and inflammatory cytokine mRNAs was significantly upregulated in myotubes isolated from the contact cocultures compared with those from the transwell cocultures (Figure 2(c)). Similarly, expression of 4-1BBL and inflammatory cytokine mRNAs was also upregulated in Raw macrophages isolated from the contact cocultures (Figure 2(d)). The next step was to investigate whether 4-1BB/4-1BBL interaction was responsible for the inflammatory response in cocultures; we blocked this interaction with neutralizing antibody TKS-1, which specifically reacts with 4-1BBL and inhibits the 4-1BB/4-1BBL interaction. As shown in Figure 2(e), treatment of cocultures with TKS-1 reduced the production of TNF*α*, IL-6, and MCP-1 released in the culture media. Consistent with this, expression of inflammatory cytokine mRNAs was downregulated in both myotubes and Raw macrophages isolated from the cocultures treated with TKS-1 (Figures 2(f) and 2(g)).

### 3.3. Stimulation of 4-1BB Increases Inflammatory Responses in Skeletal Muscle Cells

**Figure 3:**
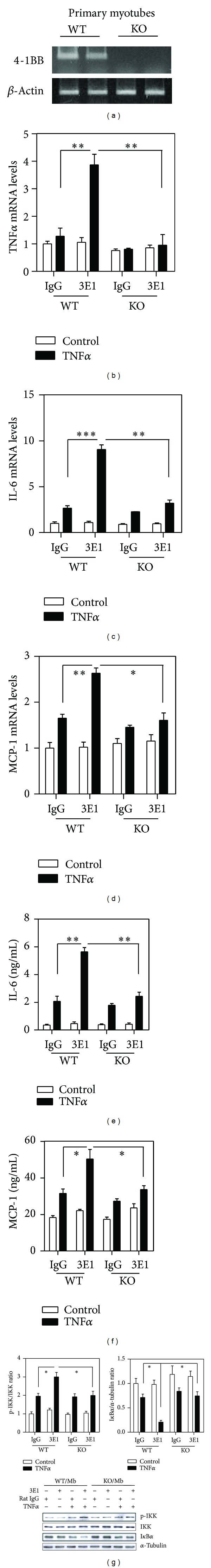
Expression of inflammatory cytokines in primary myotubes treated with 3E1. Three-day primary myotubes derived from 4-week-old WT and 4-1BB-deficient mice. (a) Representative bands of 4-1BB mRNA were determined by semiquantitative RT-PCR. Primary myotubes were pretreated with 10 ng/mL TNF*α* in DMEM containing 0.1% FBS for 12 h. Then the cells were washed twice with PBS and given 10 *μ*g/mL 3E1 or 10 *μ*g/mL rat IgG (control) in serum-free DMEM for 24 h (b–f) or 3 h (g). (b–d) qRT-PCR analysis of mRNA for the inflammatory cytokines TNF*α*, IL-6, and MCP-1. ((e) and (f)) Levels of release of inflammatory cytokines IL-6 and MCP-1 in the culture media were measured by ELISA. (g) Expression of phosphorylated IKK*α*/**β**, IKK**β**, I**κ**B*α*, and *α*-tubulin proteins was determined by Western blotting using the indicated antibodies (lower panel). Band intensities were measured densitometrically using ImageJ. Relative intensities of the bands were displayed as fold of control (upper panels). Data are means ± SEM of three independent triplicate experiments. *P < 0.01, **P < 0.01, and ***P < 0.001 significantly different between 3E1- and rat IgG-treated groups or between WT and KO groups.

To test whether 4-1BB plays a role in the increased skeletal muscle inflammation in the obese mice, we prepared primary muscle cells from mice and stimulated them with an anti-4-1BB agonistic antibody. The absence of 4-1BB in primary muscle cells derived from 4-1BB-deficient (KO) mice was confirmed by RT-PCR analysis (Figure 3(a)). As shown in Figures 3(b)–3(f), no inflammatory responses were observed in primary myotubes treated with agonistic 4-1BB antibody (3E1). However, when the primary muscle cells were pretreated with TNF*α* to mimic the inflamed microenvironment, mRNAs for inflammatory cytokines (TNF*α*, IL-6, and MCP-1) were markedly upregulated by the treatment of 3E1 (Figures 3(b)–3(d)) and the protein levels of these cytokines were also elevated (Figures 3(e) and 3(f)). These effects of 3E1 were abrogated in TNF*α*-pretreated 4-1BB-deficient myotubes (Figures 3(b)–3(f)). We next examined whether stimulation of 4-1BB on skeletal muscle cells affected the nuclear factor kappa B (NF-**κ**B) signaling pathway, which regulates inflammatory cytokine expression [5, 21]. 4-1BB stimulation of TNF*α*-pretreated WT muscle cells with 3E1 led to increased phosphorylation of I**κ**B kinase (IKK) and degradation of inhibitor of kappa B (I**κ**B*α*) (Figure 3(g)), which are upstream mediators of NF-**κ**B activation [5, 17]. The effect of 3E1 on the stimulation of IKK phosphorylation and I**κ**B*α* degradation was blunted in TNF*α*-pretreated 4-1BB-deficient myotubes (Figure 3(g)).

### 3.4. Ablation of 4-1BB Ameliorates Inflammation in the Skeletal Muscle of HFD-Fed Mice

**Figure 4:**
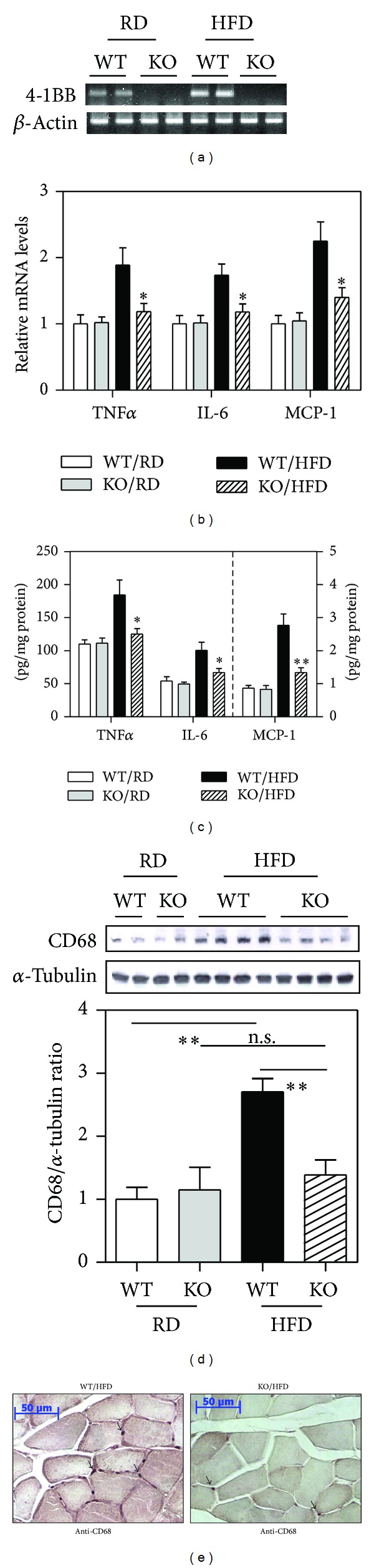
Deficiency of 4-1BB reduces inflammatory responses in skeletal muscle of HFD-fed mice. C57BL/6 wild-type (WT) and 4-1BB-deficient mice were fed a RD or HFD for 9 weeks. (a) Representative bands of 4-1BB mRNA in skeletal muscle were determined by semiquantitative RT-PCR. ((b) and (c)) TNF*α*, IL-6, and MCP-1 mRNAs and proteins were measured by qRT-PCR and ELISA, respectively. (d) Expression of CD68 and *α*-tubulin was determined by Western blotting using the indicated antibodies. (e) Immunohistochemical staining for macrophage-specific CD68 in paraffin-embedded sections of the quadriceps muscle. Arrows indicate CD68-stained cells. Magnification 200; Scale bars 50 *μ*m. Data are means ± SEM for *n* = 6. *P < 0.05 and **P < 0.01 compared with WT mice. n.s., not significant.

Since the above data indicated that increased 4-1BB expression was associated with skeletal muscle inflammation, we tested whether 4-1BB deficiency altered skeletal muscle inflammatory responses. RT-PCR analysis showed that 4-1BB mRNA was absent from the skeletal muscle of 4-1BB-deficient mice (Figure 4(a)). As shown in Figures 4(b) and 4(c), there was increased expression of inflammatory cytokines in HFD-fed compared to RD-fed WT mice, and this increase was markedly reduced in the 4-1BB-deficient mice. Similarly, no increased expression of CD68 protein, a marker of macrophages, was seen in HFD-fed 4-1BB-deficient mice (Figure 4(d)). In agreement with this, histological analysis showed that HFD-fed 4-1BB-deficient mice contained fewer CD68-positive cells than HFD-fed WT mice (Figure 4(e)). There were no differences in the levels of inflammatory cytokines and the macrophage marker between the skeletal muscle of RD-fed WT mice and 4-1BB-deficient mice (Figures 4(b)–4(d)).

## 4. Discussion

Increased skeletal muscle production of inflammatory cytokines accompanied by macrophage infiltration is a hallmark of obesity [5, 6, 22], and crosstalk between skeletal muscle cells/macrophages plays a crucial role in these inflammatory responses [5, 6] although the molecules involved remain elusive. It has been shown that cell surface molecules (receptor/ligand) mediated crosstalk is important for the onset and/or maintenance of inflammatory responses [23–25]. Here, we showed that 4-1BB and 4-1BBL expressions were upregulated in the skeletal muscle of HFD-fed mice, accompanied by increased macrophage infiltration and inflammatory cytokine production. We also found that 4-1BB expression was upregulated on muscle cells by obesity-related factors, including palmitic acid, TNF*α*, and LPS, which promote skeletal muscle cell inflammation [5, 26, 27]. These obesity-related factors are also known to induce 4-1BBL expression on macrophages [17]. In addition, the contact cocultured myotubes/macrophages induced expression of 4-1BB on myotubes and 4-1BBL on macrophages, which was accompanied by increased production of inflammatory cytokines. It has recently been reported that the interaction between cell surface molecules 4-1BB and its ligand 4-1BBL plays a crucial role in the initiation and/or maintenance of chronic inflammation induced by cell-cell contact (e.g., T cells/macrophages, endothelial cells/monocytes, and adipocytes/macrophages) [17, 23, 28]. Together, these findings suggest that 4-1BB and/or 4-1BBL participate in skeletal muscle inflammation by promoting muscle cell-macrophage interaction.

**Figure 5:**
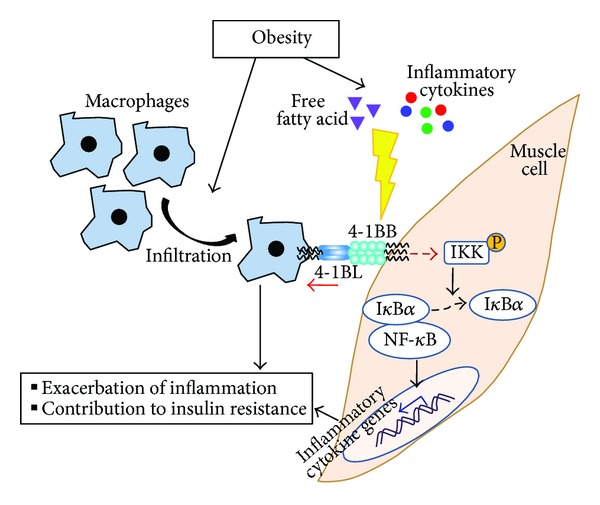
A schematic representation for the effect of 4-1BB/4-1BBL interaction on skeletal muscle inflammation. Obesity induces upregulation of 4-1BB on skeletal muscle cells, which interacts with its ligand 4-1BBL expressed on infiltrated macrophages. This interaction triggers bidirectional inflammatory signaling into both cells, leading to exacerbation of inflammatory responses and metabolic dysfunction in skeletal muscle.

It should be noted that the interaction between 4-1BB and 4-1BBL leads to bidirectional signaling [17, 19, 29]. Previously, we demonstrated that 4-1BB on adipocytes directly delivers an inflammatory signal through its interaction with 4-1BBL on macrophages and that this results in increased secretion of inflammatory cytokines from obese adipose tissue [17]. In this study, we investigated whether 4-1BB on muscle cells participates in the inflammatory crosstalk with muscle macrophages in obese skeletal muscle inflammation. Using an agonistic anti-4-1BB antibody that binds specifically to 4-1BB [19], we stimulated muscle cells and measured their inflammatory cytokine responses. Surprisingly, unlike adipocytes [17], stimulation of 4-1BB on muscle cells did not alter the expression of inflammatory cytokines in the myotubes. Because it has been shown that agonistic anti-4-1BB antibody treatment does not induce inflammatory responses in endothelial cells when 4-1BB expression is low [14], we thought that 4-1BB expression on muscle cells might not be strong enough in the noninflamed condition to lead to stimulation by the agonistic antibody. In agreement with this view, pretreatment with TNF*α* markedly upregulated 4-1BB expression, and subsequent treatment with agonistic antibody enhanced inflammatory cytokine production (TNF*α*, IL-6, and MCP-1) in muscle cells from WT mice; moreover, these changes were completely abrogated in 4-1BB-deficient muscle cells, confirming the need for 4-1BB stimulation. Furthermore, the upregulation of 4-1BB expression by TNF*α* treatment in muscle cells suggests that 4-1BB-mediated signaling may be activated in inflamed skeletal muscle in the obese condition. Indeed, 4-1BB stimulation further increased IKK activation as well as induced I**κ**B*α* degradation in WT muscle cells treated with TNF*α*. These findings suggest that 4-1BB-mediated inflammatory responses in skeletal muscle cells are mediated by activation of the NF-**κ**B signaling pathway. In addition, reverse signaling through 4-1BBL has been shown to play a crucial role in the regulation of inflammation in monocytes and macrophages [17, 28]. Given the increase of macrophages infiltration into obese skeletal muscle [5, 22], muscle cells with upregulated expression of 4-1BB may deliver an inflammatory reverse signal through 4-1BBL on macrophages. Indeed, blockade of the 4-1BB/4-1BBL interaction markedly reduced inflammatory responses in the cocultured myotubes/macrophages, and presumably both signaling through 4-1BB on myotubes and signaling through 4-1BBL on macrophages were disrupted. Taken together, these findings suggest that the 4-1BB/4-1BBL-mediated interaction between muscle cells and macrophages induces bidirectional inflammatory signaling and may be an important element of the inflammatory responses in obese skeletal muscle (Figure 5).

Next, using 4-1BB-deficient mice fed an HFD, we examined whether deficiency of 4-1BB protected against obesity-induced skeletal muscle inflammation. Indeed, deficiency of 4-1BB resulted in decreased levels of inflammatory cytokines/chemokines, including TNF*α*, IL-6, and MCP-1 in muscle. Macrophages deliver an inflammatory reverse signal through 4-1BBL that is bound to its receptor 4-1BB [30]; hence, deficiency of 4-1BB could block stimulation of 4-1BBL signaling on macrophages and also interrupt signaling through 4-1BB on muscle cells, leading to decreased cytokine production from macrophages and/or muscle cells in the skeletal muscle of HFD-fed 4-1BB-deficient mice. Importantly, since inflammatory cytokines/chemokines such as MCP-1 have a strong affinity for macrophages [31, 32], the decreased macrophages infiltration into skeletal muscle of HFD-fed 4-1BB-deficient mice could result from the reduced expression of this factor. Furthermore, given that increased inflammatory cytokine production accompanied by macrophage infiltration into skeletal muscle is closely associated with both local and systemic insulin resistance [5, 22], the observation of increases in both skeletal muscle and systemic insulin sensitivity in HFD-fed 4-1BB-deficient mice [16] may be at least partly attributed to lowered inflammatory responses in the skeletal muscle. Collectively, these results indicate that the 4-1BB/4-1BBL interaction plays a crucial role in obesity-induced skeletal muscle inflammation.

In conclusion, our data demonstrate that 4-1BB/4-1BBL interaction may aggravate inflammatory responses in inflamed skeletal muscle in obesity by triggering bidirectional inflammatory signaling in muscle cells and macrophages. Disruption of the 4-1BB/4-1BBL interaction reduces the inflammation resulting from crosstalk between muscle cells and macrophages and protects against HFD-induced inflammatory responses in skeletal muscle. Both 4-1BB and 4-1BBL could be useful targets for preventing obesity-induced skeletal muscle inflammation.

## Figures and Tables

**Table 1:** Mouse primers used for qRT-PCR analysis.

Gene	Forward primer (5′→3′)	Reverse primer (5′→3′)
4-1BB	CTCTGTGCTCAAATGGATCAGGAA	TGTGGACATCGGCAGCTACAA
4-1BBL	CCTGTGTTCGCCAAGCTACTG	CGGGACTGTCTACCACCAACTC
TNF*α*	AAGCCTGTAGCCCACGTCGTA	GGCACCACTAGTTGGTTGTCTTTG
IL-6	CCACTTCACAAGTCGGAGGCTTA	GCAAGTGCATCATCGTTGTTCATAC
MCP-1	GCATCCACGTGTTGGCTCA	CTCCAGCCTACTCATTGGGATCA
*β*-actin	CATCCGTAAAGACCTCTATGCCAAC	ATGGAGCCACCGATCCACA

## References

[1] Lumeng CN, Saltiel AR (2011). Inflammatory links between obesity and metabolic disease. *Journal of Clinical Investigation*.

[2] Hotamisligil GS (2006). Inflammation and metabolic disorders. *Nature*.

[3] Valerio A, Cardile A, Cozzi V (2006). TNF-*α* downregulates eNOS expression and mitochondrial biogenesis in fat and muscle of obese rodents. *Journal of Clinical Investigation*.

[4] Tang K, Wagner PD, Breen EC (2010). TNF-*α*-mediated reduction in PGC-1*α* may impair skeletal muscle function after cigarette smoke exposure. *Journal of Cellular Physiology*.

[5] Varma V, Yao-Borengasser A, Rasouli N (2009). Muscle inflammatory response and insulin resistance: synergistic interaction between macrophages and fatty acids leads to impaired insulin action. *The American Journal of Physiology*.

[6] Patsouris D, Li P-P, Thapar D, Chapman J, Olefsky JM, Neels JG (2008). Ablation of CD11c-positive cells normalizes insulin sensitivity in obese insulin resistant animals. *Cell Metabolism*.

[7] Koves TR, Ussher JR, Noland RC (2008). Mitochondrial overload and incomplete fatty acid oxidation contribute to skeletal muscle insulin resistance. *Cell Metabolism*.

[8] Du M, Yan X, Tong JF, Zhao J, Zhu MJ (2010). Maternal obesity, inflammation, and fetal skeletal muscle development. *Biology of Reproduction*.

[9] Hurtado JC, Kim SH, Pollok KE, Lee ZH, Kwon BS (1995). Potential role of 4-1BB in T cell activation: comparison with the costimulatory molecule CD28. *Journal of Immunology*.

[10] Kim D-H, Chang W-S, Lee Y-S (2008). 4-1BB engagement costimulates NKT cell activation and exacerbates NKT cell ligand-induced airway hyperresponsiveness and inflammation. *Journal of Immunology*.

[11] Vinay DS, Kwon BS (1998). Role of 4-1BB in immune responses. *Seminars in Immunology*.

[12] Takahashi C, Mittler RS, Vella AT (1999). Cutting edge: 4-1BB is a bona fide CD8 T cell survival signal. *Journal of Immunology*.

[13] Cheung CTY, Deisher TA, Luo H (2007). Neutralizing anti-4-1BBL treatment improves cardiac function in viral myocarditis. *Laboratory Investigation*.

[14] Jeon HJ, Choi J-H, Jung I-H (2010). CD137 (4-1BB) deficiency reduces atherosclerosis in hyperlipidemic mice. *Circulation*.

[15] Nguyen Q-T, Ju S-A, Park S-M (2009). Blockade of CD137 signaling counteracts polymicrobial sepsis induced by cecal ligation and puncture. *Infection and Immunity*.

[16] Kim C-S, Kim JG, Lee B-J (2011). Deficiency for costimulatory receptor 4-1BB protects against obesity-induced inflammation and metabolic disorders. *Diabetes*.

[17] Tu TH, Kim CS, Goto T, Kawada T, Kim BS, Yu R (2012). 4-1BB/4-1BBL interaction promotes obesity-induced adipose inflammation by triggering bidirectional inflammatory signaling in adipocytes/macrophages. *Mediators of Inflammation*.

[18] Kwon BS, Hurtado JC, Lee ZH (2002). Immune responses in 4-1BB (CD137)-deficient mice. *Journal of Immunology*.

[19] Shuford WW, Klussman K, Tritchler DD (1997). 4-1BB costimulatory signals preferentially induce CD8+T cell proliferation and lead to the amplification in vivo of cytotoxic T cell responses. *Journal of Experimental Medicine*.

[20] Rando TA, Blau HM (1994). Primary mouse myoblast purification, characterization, and transplantation for cell-mediated gene therapy. *Journal of Cell Biology*.

[21] Jové M, Planavila A, Sánchez RM, Merlos M, Laguna JC, Vázquez-Carrera M (2006). Palmitate induces tumor necrosis factor-*α* expression in C_2_C_12_ skeletal muscle cells by a mechanism involving protein kinase C and nuclear factor-*κ*B activation. *Endocrinology*.

[22] Schenk S, Saberi M, Olefsky JM (2008). Insulin sensitivity: modulation by nutrients and inflammation. *Journal of Clinical Investigation*.

[23] Olofsson PS, Söderström LÅ, Wågsäter D (2008). CD137 is expressed in human atherosclerosis and promotes development of plaque inflammation in hypercholesterolemic mice. *Circulation*.

[24] Haga T, Suzuki J-I, Kosuge H (2009). Attenuation of experimental autoimmune myocarditis by blocking T cell activation through 4-1BB pathway. *Journal of Molecular and Cellular Cardiology*.

[25] Fiocchi C (1997). Intestinal inflammation: a complex interplay of immune and nonimmune cell interactions. *The American Journal of Physiology*.

[26] Frisard MI, McMillan RP, Marchand J (2010). Toll-like receptor 4 modulates skeletal muscle substrate metabolism. *The American Journal of Physiology*.

[27] Li Y-P, Chen Y, John J (2005). TNF-*α* acts via p38 MAPK to stimulate expression of the ubiquitin ligase atrogin1/MAFbx in skeletal muscle. *The FASEB Journal*.

[28] Kwon B (2009). CD137-CD137 ligand interactions in inflammation. *Immune Network*.

[29] Drenkard D, Becke FM, Langstein J (2007). CD137 is expressed on blood vessel walls at sites of inflammation and enhances monocyte migratory activity. *The FASEB Journal*.

[30] Schwarz H (2005). Biological activities of reverse signal transduction through CD137 ligand. *Journal of Leukocyte Biology*.

[31] Lu H, Huang D, Ransohoff RM, Zhou L (2011). Acute skeletal muscle injury: CCL_2_ expression by both monocytes and injured muscle is required for repair. *The FASEB Journal*.

[32] Kanda H, Tateya S, Tamori Y (2006). MCP-1 contributes to macrophage infiltration into adipose tissue, insulin resistance, and hepatic steatosis in obesity. *Journal of Clinical Investigation*.

